# Response to Letter to the Editor: “Can back-wall cystic figures of thyroid nodules predict benignity?”

**DOI:** 10.1530/ETJ-26-0108

**Published:** 2026-04-16

**Authors:** Jean-Guillaume Marchand, Marie Bienvenu-Perrard, Agnes Rouxel, Cécile Ghander, Camille Buffet, Gilles Russ

**Affiliations:** ^1^ Thyroid and Endocrine Tumors Department, La Pitié-Salpêtrière Hospital, Sorbonne University, GRC No. 16, Paris, France; ^2^ Centre of Pathology and Radiology, Paris, France; ^3^ Nuclear Medicine Department, Cochin Hospital, Paris, France; ^4^ Nuclear Medicine Department, Avicenne Hospital, Bobigny, France

**Keywords:** back-wall cystic figure, thyroid nodule, ultrasound, EU-TIRADS, hyperechoic foci

Dear Editor,

We sincerely thank the authors for careful reading of our work (1) and their insightful comments (2).

However, we believe that an important clarification regarding the scope of our study is necessary. Our analysis strictly concerns back-wall cystic figures (BWCFs) as defined in the ETA 2023 lexicon (3), namely bright echogenic lines and punctate granules in the dorsal wall of cystic areas caused by posterior enhancement. This definition inherently excludes macrocystic, necrotic, or predominantly cystic lesions.

Furthermore, our study is deliberately restricted to EU-TIRADS 4 nodules. In this context, several of the entities mentioned by the authors in this letter (such as cystic variants of papillary thyroid carcinoma, lymphoma, or metastatic lesions) typically present with ultrasound features of high suspicion (e.g. microcalcifications, irregular margins, or marked hypoechogenicity) and would therefore be classified as EU-TIRADS 5 rather than EU-TIRADS 4 according to the current guidelines. As such, their inclusion in the present discussion is not directly applicable to the population we specifically investigated.

We also note that hyperechoic spots of uncertain significance (as described in the ETA 2017 guidelines) (4) may occasionally appear in association with BWCFs. In such cases, these hyperechoic spots of uncertain significance should be considered as microcalcifications until proven otherwise. Similarly, if BWCFs were to coexist with true microcalcifications, true microcalcifications would predominate in the classification, resulting in an EU-TIRADS 5.

Regarding the underlying mechanism, we agree that BWCFs are related to acoustic phenomena and acknowledge the author emphasis on attenuation and sound transmission. However, this physical explanation does not alter the practical issue, which is the reproducible identification of a specific imaging pattern. Our objective was not to establish a histopathological correlate, but to assess the value of this well-defined sonographic sign for risk stratification within an EU-TIRADS category.

Finally, while posterior acoustic enhancement may indeed be observed in a variety of malignant or non-neoplastic conditions, these situations typically present with additional structural or clinical features. They should not be conflated with the strict microcystic BWCF pattern used in our inclusion criteria.

We therefore maintain that our results are valid within this intentionally well-defined framework. They show that the presence of BWCFs, as strictly defined and illustrated in Fig. 1, in EU-TIRADS 4 nodules is associated with a significantly lower malignancy risk, close to that of EU-TIRADS 3 nodules, while their absence is linked to a higher rate of indeterminate cytology. These findings support the importance of distinguishing BWCFs from other hyperechoic foci to refine risk stratification.

## Declaration of interest

The authors declare that there is no conflict of interest that could be perceived as prejudicing the impartiality of the work reported.

## Funding

This work did not receive any specific grant from any funding agency in the public, commercial, or not-for-profit sector.
